# To the Editors of the Medical and Physical Journal

**Published:** 1810-06

**Authors:** 


### *To the Editors of the Medical and Physical Journal.*

GENTLEMEN,

ALLOW me, through the medium of your valuable Work, to suggest a few remarks on the subject of uterine hæmorrhage, as far as it regards the treatment of those cases where the placenta is attached over the os uteri. I am the more induced to trouble you with my ideas on the subject, as two papers have lately appeared in your Journal expressly on the subject, in which a contrariety of opinion is manifested. In midwifery, as in all the other branches of the profession, rash temerity is as much to be condemned as supine inertness, both are equally unjustifiable in cases of danger.

Mr. Davis, in his paper, justly observes, that in other cases, the practitioner will have plenty of time to deliberate upon the best mode of treatment to be adopted, but in those alarming cases to which he alludes no time is to be lost; his observation is certainly highly judicious and deserves attention, and I do certainly think it much safer to lean on this *side*, than to procrastinate and delay your exertions. Mr. Spencer seems to think this *bad advice* for junior practitioners, and likely to produce rash and undetermined practice; he advises a consultation to be called. But let it be remembered that this is not always practicable; if it was, it certainly is most adviseable, and ought to be resorted to. Danger in those cases of attachment of the placenta over the os uteri, is always increased in proportion to its dilatation, consequently it becomes highly necessary to deliver as early as possible, as every increase of dilatation produces an increase of hæmorrhage, and consequent danger. There can be no determined rule laid down in these cases respecting the safety in waiting for the dilatation; for one woman shall bear the loss of twice as much blood as another, with no more injury to the constitution; but still it is of consequence to prevent as much as possible a great loss of blood, and the only possible means of doing that is by *early delivery*; by waiting, the patient becomes *more* and *more* exhausted from profuse hæmorrhage, and consequently is ill adapted to undergo the fatigue of an operation. By thus urging promptitude and *decision*, I by no means advocate *rashness*, that is highly reprehensible. Before an operation is performed (whether in surgery or midwifery) the grounds upon upon which it is done ought to be well understood, and the practitioner satisfied in his own mind that it is necessary; if so, then surely there can be no blame attached to performing it at once, without any further hesitation and delay. Another point of some consequence in these cases, is the state of the os uteri, not only to ascertain how *much* it is dilated, but whether it is dilatable; if it is in that state as to admit of easy dilatation without much force, there can be no *doubt* as to the propriety of proceeding in the operation; if, on the contrary, it is in a state of rigid contraction, I should think we may safely wait till dilatation begins to take place. As I observed before, hæmorrhage can only increase in proportion as the dilatation goes on; if the patient is of a full plethoric habit of body, would it not be adviseable to bleed, in order to induce a relaxation of the system?

These few remarks are penned not with a view to canvas the merits of either of your respectable Correspondents, but with a hope that other contributors to your Journal, may be induced to offer their observations upon so important a subject.

I am, &c.

OBSTETRICUS.

